# Age and pain score before gastrointestinal endoscopies in children are predictors for post procedure pain

**DOI:** 10.1186/s12876-020-01546-y

**Published:** 2020-11-26

**Authors:** Tut Galai, Anat Yerushalmy-Feler, Nathan P. Heller, Amir Ben-Tov, Yael Weintraub, Achiya Amir, Hadar Moran-Lev, Lilach Zac, Shlomi Cohen

**Affiliations:** 1 Pediatric Gastroenterology Unit, “Dana-Dwek” Children’s Hospital, Tel Aviv Medical Center, 6 Weizmann Street, 6423906 Tel Aviv, Israel; 2 Department of Anesthesiology and Critical Care, Tel Aviv Sourasky Medical Center, Tel Aviv, Israel; 3 Sackler Faculty of Medicine, Tel Aviv University, Tel Aviv, Israel

**Keywords:** Biopsy, Children, Endoscopy, Gastrointestinal, Pain, Predictors

## Abstract

**Background:**

Gastrointestinal endoscopy may be associated with pain and anxiety. Predictors for high pain scores after endoscopies in children are not known. The aim of our study was to identify risk factors for prolonged recovery and higher pain scores after gastrointestinal endoscopy in children.

**Methods:**

All the children that were electively admitted for gastrointestinal endoscopies were included. We retrospectively collected demographic, clinical and endoscopic data as well as information on the recovery process. A numerical rating scale and the Faces, Legs, Activity, Cry, and Consolability Scale were used for pain scoring.

**Results:**

During the study period (01/2016–10/2016), 284 children (median age 10.7 years, interquartile range 6.7–14.8) were recruited. In a univariate analysis, older age, higher pre-procedure pain scores, longer procedure durations, higher number of biopsies and longer recovery duration were associated with higher post-procedure pain scores. In a multivariate analysis higher pain scores before the procedure (OR 12.42, 95% CI 3.67–42, *P* < 0.001) and older age (OR 1.016, 95% CI 1.007–1.025, *P* < 0.001) were associated with higher pain scores after the procedure. Children with a higher pain score before the procedure also had a longer recovery period (OR 5.28, 95% CI (1.93–14.49), *P* = 0.001).

**Conclusion:**

Older age and higher pain score before the procedure were identified as predictors for higher pain score after pediatric gastrointestinal endoscopies. Children with these risk factors should be identified before the procedure in order to personalize their post-procedure management.

## Background

Gastrointestinal (GI) endoscopies have been playing an increasing role in the diagnosis and management of several GI conditions. These procedures may be associated with discomfort, pain and anxiety, especially in the pediatric population.

Although GI endoscopies are frequently performed in daily practice, there are no data regarding the post-procedure recovery in the pediatric population. Lee et al. [1] reported that female gender and longer duration of colonoscopy increased the likelihood of post-procedure abdominal pain in the adult population. Other risk factors for prolonged pain after endoscopic examination were irritable bowel syndrome and conscious sedation [1]. In addition, children may experience a host of negative experiences related to medical procedures including fear, anxiety and sensory pain. Some of them, especially young children [2], are unable to differentiate anxiety from pain, and this can also affect post-procedure pain scores.

Understanding the risk factors for high pain scores during recovery from GI endoscopies will facilitate the targeting of more accurate and personalized preparation for the child and family. Our hypothesis was that pain and prolonged recovery after GI endoscopies can be predicted in advance. The objective of this study was to evaluate the recovery process, including recovery duration and pain, of children after upper and lower GI endoscopies under sedation and to identify risk factors for a high pain score during recovery.

## Methods

### Patients

In this retrospective study, we included all male and female children (age 1–18 years) that were electively admitted for upper and/or lower GI endoscopies at the Pediatric Gastroenterology Unit, “Dana-Dwek” Children’s Hospital, Tel Aviv Sourasky Medical Center, between January 2016 and October 2016. Our hospital is a tertiary referral center for pediatric gastroenterology. The patients were identified through the local electronic health records database according to admission codes “upper GI endoscopy” and “lower GI endoscopy”. Children that underwent the procedure not at the Pediatric Gastroenterology Unit (i.e. at the pediatric intensive care unit due to body weight < 10 kg or because of high risk for sedation) were not included due to different sedation and monitoring protocols. We excluded children that underwent the procedure in an acute setting during hospitalization, those that needed intubation and ventilation, as well as those with significant neurologic or psychiatric conditions that could affect the recovery process. For gas insufflation, we used ambient air for all procedures. The first procedure was taken for analysis when children underwent multiple endoscopies. Upper GI endoscopies, lower GI endoscopies and upper plus lower GI endoscopies were analyzed separately. The study was approved by the Institutional Review Board of Tel Aviv Sourasky Medical Center (TLV-0144-17).

### Sedation during the procedure

Deep sedation during endoscopy was provided by intravenous propofol by a qualified anesthesiologist. Premedication with intravenous midazolam or induction with inhaled gas (sevoflurane and nitrous oxide) was provided in some cases at the discretion of the anesthesiologist.

### Patient assessment

A trained nurse evaluated the patients upon arrival and before any procedure and monitored them in the recovery room. This was the same nurse in all procedures, in the pre and post procedure assessment. This assessment was a part of the regular clinical assessment that was recorded in the medical record. Vital signs and pain scores were taken routinely 15 min before the procedure and every 10 min during recovery while the child was under sedation and while awake. We used the 0 to 10 Numerical Rating Scale (NRS-11), which is a scale for pain assessment used for a variety of conditions (Supplementary file 1). This scale assesses self-reported pain intensity in children [3, 4] and is applicable for children 8 years of age and older. Another scale is the Faces, Legs, Activity, Cry, and Consolability (FLACC) scale, which is a behavioral observational pain assessment scales in multiple settings for younger children or children with cognitive impairment ([5, 6] Supplementary file 1). The NRS-11 was used for children 8 years of age and older. For children below 8 years, the FLACC scale was used. For the analysis, we combined the two scales. The maximal awake pain score was used for statistical analysis. Children were categorized into three groups according to their post-procedure pain score: 0–2 = no/mild pain, 3–5 = moderate pain and 6+ = severe pain [7]. Recovery duration was defined as time from end of the procedure until discharge from hospital, and prolonged recovery was defined as the highest quartile of recovery duration.

### Data collection

We retrospectively reviewed the patients’ records and collected demographic data consisting of age and gender, symptoms, type and duration of procedure, number of biopsies, macroscopic and microscopic findings, years of seniority of the gastroenterologist or anesthesiologist, type and dose of sedative agent, duration of the recovery process, vital signs, pain scores, and location of pain.

### Statistical analysis

Categorical variables were described as frequency and percentages. Continues variables were evaluated for normal distribution using histograms and Q-Q plots and reported as median and interquartile range (IQR). Categorical variables were compared between the studied outcomes using the chi-square test or Fisher’s exact test. Continuous variables were compared using the Kruskal Wallis or Mann Whitney test. Variables that were significantly associated with the outcome according to the univariate analysis were further adjusted to age and gender using multinomial logistic regression or binary logistic regression. All statistical tests were two-sided and a *P* value < 0.05 was considered statistically significant. SPSS was used for all statistical analyses (IBM SPSS statistics, version 22, 2013, IBM CORP. Armonk, NY, USA).

## Results

### Demographic and clinical data

A total of 380 children underwent 410 GI endoscopic procedures. Seventy-three of the cases were excluded due to missing data, and 23 were excluded because the procedure was performed in an acute setting. Thirty procedures were multiple endoscopies, leaving a total of 284 procedures that were performed on 284 children for final analysis. Of these children, 117 (41.2%) were males and 167 (58.8%) were females, with a median age of 10.7 years and range of 1.5–18 years [interquartile range (IQR) of 6.7–14.8 years]. An upper GI endoscopy was performed in 210 cases (73.9%), lower GI endoscopy in 16 (5.6%), and both upper and lower GI endoscopies in 58 (20.4%). Therapeutic procedures (mainly polypectomy) were performed in six cases (2.4%).

The main indications for the GI endoscopy were abdominal pain (*n* = 117, 41.2%), suspected celiac disease (*n* = 100, 35.2%), investigation of diarrhea (*n* = 30, 10.6%) and polyposis surveillance (*n* = 21, 7.4%). Other indications for endoscopy were rectal bleeding, vomiting, failure to thrive, iron deficiency and dysphagia.

The main histological findings were celiac disease (*n* = 82, 28.9%), gastritis (*n* = 78, 27.5%), inflammatory bowel disease (*n* = 25, 8.8%) and normal (*n* = 88, 31%).

Sedation included propofol in all children in addition to gas induction in 34 (12%) and midazolam in 51 (18%). The median (IQR) procedure duration was 9 (6–32) minutes and the median (IQR) recovery duration was 2.1 (1.8–2.7) hours (Table 1).

**Table 1:** Demographic and clinical data by post procedure pain score

	Total *N* = 284	No to mild Pain 0–2 *N* = 199	Moderate Pain 3–5 *N* = 50	Severe Pain 6+ *N* = 35	P
Age years, median (IQR)	10.7 (6.7–14.8)	9.5 (5.6–14)	12.4 (8.7–14.9)	14.4 (10.5–16)	< 0.001
Gender:					0.485
Female	167 (58.8%)	115 (57.8%)	33 (66%)	19 (54.3%)	
Male	117 (41.2%)	84 (42.2%)	17 (34%)	16 (45.7%)	
Procedure:					< 0.001
Gastroscopy	210 (73.9%)	164 (82.4%)	30 (60%)	16 (45.7%)	
Colonoscopy	16 (5.6%)	10 (5%)	3 (6%)	3 (8.6%)	
Both	58 (20.4%)	25 (12.6%)	17 (34%)	16 (45.7%)	
Pre-procedural pain:					< 0.001
0–2	237 (83.4%)	174 (87.4%)	43 (86.0%)	20 (57.1%)	
3–5	22 (7.7%)	11 (5.5%)	5 (10.0%)	6 (17.1%)	
6+	25 (8.8%)	14 (7.0%)	2 (4.0%)	9 (25.7%)	
Procedure duration (min)	9 (6–32)	7 (6–14)	13 (6–46.5)	35 (7.5–58.2)	< 0.001
Number of biopsies	3 (2–4)	3 (2–3)	3 (2–5.2)	5 (3–8)	< 0.001
Location of pain:					< 0.001
Abdomen	66 (57.9%)	10 (29.4%)	28 (63.6%)	28 (77.7%)	
Hand	26 (22.8%)	22 (64.7%)	3 (6.8%)	1 (2.7%)	
Head	18 (15.8%)	2 (5.8%)	12 (27.2%)	4 (11.1%)	
Throat	4 (3.5%)	0 (0%)	1 (2.2%)	3 (8.3%)	
Recovery duration (h)	2.1 (1.8–2.7)	2.1 (1.8–2.5)	2.2 (1.7–2.7)	2.6 (1.9–3.3)	0.018

### Post-procedure pain

A total of 170 children (59.8%) had no post-procedural pain, 29 children (10.2%) had mild pain (a score of 1–2), 50 children (17%) had moderate pain (a score of 3–5) and 35 children (12.3%) had severe pain (a score of 6 and above) (Table 1). Of 114 patients (40.1%) with post-procedure pain, in 66 (57.9%) the pain originated in the abdomen and in 26 (22.8%) in the intravenous line, while 18 (15.8%) complained of headache, and 4 (3.5%) of throat pain.

#### Risk factors for high post-procedure pain scores

Children with higher post-procedure pain scores were significantly older (significant results for severe and moderate pain compared to mild, *P* = 0.005), had higher pre-procedure pain scores (significant results for severe pain compared to moderate and mild, *P* = 0.003 and *P* < 0.001, respectively), longer procedure durations, higher numbers of biopsies and longer recovery durations (Table 1). While in 188 (66.2%) children the level of pre and post-procedure pain was similar, in 27 (9.5%) children the pre-procedure pain was more severe compared to the post-procedure pain, and in 69 (24.3%) children the pain was escalated after the procedure (Table 1).

There was no relation between pain score and gender, vital signs, the experience of the gastroenterologist or anesthesiologist, endoscopic findings, dose of propofol and the use of other sedation agents (midazolam or inhaled gas) in addition to propofol.

In a multivariate analysis adjusted for age, gender and type of procedure, older age and higher pain score before the procedure were associated with higher post-procedure pain scores (Table 2).

**Table 2:** Multivariate analysis^a^ for higher post-procedure pain scores

		Adjusted OR	95% CI	P
All endoscopies (*N* = 284)	Age	1.016	1.007–1.025	< 0.001
	Pain score before the procedure	12.42	3.67–42	< 0.001
Upper GI endoscopies (*N* = 210)	Age	1.015	1.004–1.026	0.008
	Pain score before the procedure	8.097	1.990–32.939	0.003
	Post-procedure abdominal pain	5.2	1.34–20.4	0.017

The variables that were included in the multivariate analysis were pre-procedure pain scores, procedure duration, number of biopsies, endoscopic findings, dose of propofol/midazolam

^a^Every variable was adjusted for age, gender and type of procedure

#### Risk factors in children underwent upper GI endoscopy

A total of 210 children underwent upper GI endoscopy. Their median (IQR) age was 9.86 (6.09–13.28) years, and 130 (61.9%) were males. A multivariate analysis of these cases revealed that older age, higher pain score before the procedure, and post-procedure abdominal pain were associated with higher post-procedure pain scores (Table 2).

#### Risk factors for high post-procedure abdominal pain scores

One-hundred and fourteen patients (40.1%) had post-procedure pain, and 66 of them (57.9%) originating in the abdomen. When compared to 218 patients who had no post-procedural pain, or pain in other locations, or pain in no specified location, those with abdominal pain were older [13.6 (9.5–15.2) years compared to 9.9 (5.8–14.2), *P* < 0.001)], had frequent diarrhea as a symptom (22.7% compared to 6.9%, *P* < 0.001), had a longer procedure [32 (10–55.7) minutes compared to 7 (6–14) minutes, *P* < 0.001)], and had a higher number of biopsies [4 (3–8) compared to 3 (2–3), *P* < 0.001)]. The children with post-procedure abdominal pain had higher post-procedure pain scores [42.4% compared to 0 with 6+ pain score, *P* < 0.001,) and higher pre-procedure pain scores (19.4% compared to 6.3% with 6+ pain score, *P* < 0.001) compared to children with pain in other locations.

### Recovery time

A longer procedure (*P* = 0.002) and a higher post-procedure pain score (*P* = 0.021) were associated with a prolonged recovery time. A multivariate analysis adjusted for age and gender revealed that the patients with a higher pre-procedure pain score had a prolonged recovery time (OR 5.28, 95% CI 1.93–14.49, *P* = 0.001).

## Discussion

Our study evaluated the recovery process after GI endoscopies in children for what we believe to be the first time. The results revealed several predictors for higher pain scores and longer recovery duration. A univariate analysis demonstrated that children with higher post-procedure pain scores had higher pre-procedure pain scores, longer-duration procedures, higher numbers of biopsies, and longer recovery time. The multivariate model showed that higher pre-procedure pain scores were positively correlated with higher post-procedure pain scores. Higher pre-procedure pain scores and longer durations of the procedures were associated with longer time to recovery. Older age was significantly related to higher post-procedure pain scores, but not to a level of clinical significance.

While some factors that were associated with a higher pain score during recovery in our study could be anticipated, such as procedure duration, others, such as older age, were unexpected. This latter finding may be explained by a higher cognitive ability of older children to communicate their pain. A behavioral observation scale (FLACC) was used for pain scoring in children under the age of 7 years in our unit. Its limitations in post-procedure pain assessment, however, have been recently reported [8]. Older age may actually be an advantage where pain-relieving methods are involved. Kristensen et al. [9] showed that the presence of a hospital clown during venipuncture promoted a pain-relieving effect in children older but not younger than 6 years of age.

We found that a higher score of pre-procedure pain was an independent risk factor for post-procedural pain. Although our study evaluated pain rather than anxiety, this finding could be explained by a positive correlation between anxiety and pain. This is a common finding in the clinical setting and pain-related anxiety can increase perceived pain intensity as shown in previous studies. Pre-procedure pain can be related to fear and anxiety associated with the procedure itself and might have an impact on pain perception immediately after the procedure. Previous studies suggested that children under the age of 8 years were unable to differentiate between sensory and affective components of painful experiences [2]. This could be explained by the biological interaction between the physiological effects of anxiety and pain perception, mainly by activation of adrenergic response which mediates hyperalgesia [10]. The relation between pre- and post-procedure pain is consistent with previous studies that showed a correlation between pre-and post-procedure anxiety and pain in the adult population [11, 12]. On the contrary, those patients may recover from anesthesia with relief of anxiety and pain accordingly, and by that, it is not obvious that children with high pre-procedure pain will necessarily have high post-procedure pain. Still, this finding is clinically meaningful since it is not obvious that children with high pre-procedure pain will necessarily have high post-procedure pain.

The findings of a positive correlation between the duration of the procedure and the post-procedure pain score are similar to those of Lee et al. [1] who showed that the duration of colonoscopy procedures was positively correlated to the severity of post-procedure abdominal pain in adults. The authors suggested that the total duration of procedure was positively correlated with the amount of insufflated air required to maintain luminal visualization. The more severe pain in colonoscopies compared to gastroscopies is probably also related to the longer duration of the procedure. The association between the numbers of biopsies to the pain score might also originate in the duration of procedure. The correlation of a longer recovery to a longer procedure and a higher pain score after the procedure again reflects the central role of pain in the process of recovery.

Although we anticipated vital signs to be influenced by pain, we did not find any correlation between post procedural pain and vital signs. Our results are in line with previous studies, that have shown no association between vital signs and self-reported acute pain intensity [13, 14]. Nevertheless, no correlation was found between pain and macroscopic as well as microscopic diagnosis (except celiac disease for upper GI endoscopies). In addition, no relation was found to the experience of the endoscopist and anesthesiologist. This observation supports our assumption that post procedure pain was not originated from the procedure itself or the gastrointestinal condition, but was affected by other factors that influence pain perception, such as anxiety or visceral hyperalgesia sensation, which is part of the pathogenesis of functional abdominal pain [15]. Our results are in agreement with the report of Allen et al. [16], observing that pain reported post colonoscopy was not more likely among patients diagnosed with colorectal disease or malignancy.

In our cohort, abdominal pain was found to be the most frequent and troublesome pain of the patients. Children with post-procedure abdominal pain had significantly higher pain scores, even before the procedure, and had a longer procedure with a higher number of biopsies compared to children with pain in other locations. The post procedure abdominal pain may originate in the underlying GI condition or in the procedure itself. It also reassures our observation that most children with significant post procedure pain will benefit from pain-relieving methods focusing or directed to the abdomen.

This is the first pediatric study that investigated the elements of recovery after GI endoscopies in children. We described the pain levels after these procedures and their relation to demographic and clinical factors. Predicting post procedure pain level and recovery duration enables preparation in advance. For patients and families – to receive an appropriate explanation about post procedure pain in order to better prepare, mentally and physically, to the procedure. This is also important for caregivers and healthcare facilities, in terms staff availability, planning the occupancy of the recovery room and making arrangements for hospitalization if needed. In addition, children with risk factors for higher post procedural pain and longer recovery could be prioritized for admission in order to perform the endoscopy in early morning hours in order to enable them to recover in day time setting. Treating children at risk with preventive analgesic medication is another possibility to reduce post procedure pain, as well as methods like medical clowns and other anxiolytic activities or analgesics. These and other methods to reduce pre- and post-procedure anxiety and pain have been shown to be effective [17–19]. Prospective interventional randomized control studies are needed to investigate the benefits of early recognition and pre-procedure intervention in children at risk.

Our study is limited by its retrospective nature. Documentation of symptoms in patients’ charts was performed, however, according to the same protocol before the procedure and during recovery, and by the same trained nurse. Almost one-fifth of the patients were excluded due to missing data, however the baseline demographic and clinical characteristics of children that were excluded were not significantly different from the patients that were included. The exclusion of patients that needed intubation and ventilation was done to enable the best representation of the majority of patients that are electively invited to perform GI endoscopies in our unit, and to permit the most accurate and reliable documentation of pain. In addition, since relatively few children had colonoscopies, we reanalyzed the children that underwent upper GI procedures separately from the others. The combination of pain scores from different pain scales could be considered another limitation, however, this enabled us to include children in a wide range of ages and therefore represent a more “real-life” population. The wide range of ages in the study cohort could be considered as another limitation since it may potentially affect procedural pain and the recovery process. However, although age was found to be an independent risk factor for high pain score, the adjusted odds ratio was close to 1. In addition, since the pre and post procedure pain was reported by the same nurse, another potential limitation is “reporting bias” influencing pain ratings. For gas insufflation, in 2016 we used ambient air for all procedures. Many units use now carbon dioxide insufflation [20], that might have a positive effect on pain. Therefore, our results and conclusion may be appropriate to units that use air insufflation. Lastly, it is always difficult to separate between pain and anxiety. No anxiety scales were used in this study.

## Conclusions

In conclusion, we identified pre-procedure pain as a predictor for higher pain scores after GI endoscopies in children. Identification of patients at high risk for a high pain score during recovery before they undergo a procedure will enable care providers to improve the management of the child and reduce the risk for pain and anxiety.

## Supplementary information

**Additional file 1.** The pain assessment scales: Numerical Rating Scale (NRS-11) and Faces, Legs, Activity, Cry, and Consolability (FLACC) scale.

## Data Availability

The datasets used and/or analyzed during the current study are available from the corresponding author on reasonable request.

## Abbreviations

GI: Gastrointestinal
NRS: Numerical Rating Scale
FLACC: Faces, Legs, Activity, Cry, and Consolability
IQR: Interquartile range
