# Supplementary material for: Age and pain score before gastrointestinal endoscopies in children are predictors for post procedure pain
Source: BMC Gastroenterol. 2020 Nov 26;20:400. doi: 10.1186/s12876-020-01546-y (PMC7689973; doi:10.1186/s12876-020-01546-y)
Supplement: Supplementary file 1 — Additional file 1. The pain assessment scales: Numerical Rating Scale (NRS-11) and Faces, Legs, Activity, Cry, and Consolability (FLACC) scale. [file 12876_2020_1546_MOESM1_ESM.docx]

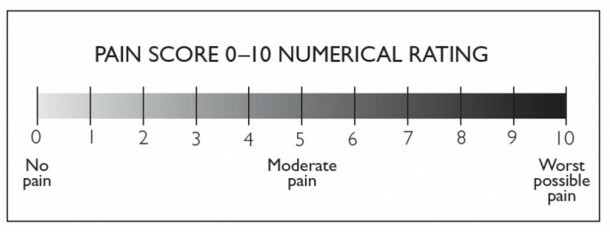


<https://www.physio-pedia.com/Numeric_Pain_Rating_Scale>

Kochman A, Howell J, Sheridan M et al. Reliability of the Faces, Legs, Activity, Cry, and Consolability Scale in assessing acute pain in the pediatric emergency department. Pediatr Emerg Care. 2017; 33: 14-17 (Reference 6)
